# MicroRNA与肺癌顺铂耐药相关性研究进展

**DOI:** 10.3779/j.issn.1009-3419.2014.03.15

**Published:** 2014-03-20

**Authors:** 洋 李

**Affiliations:** 1 300070 天津，天津医科大学 Tianjin Medical University, Tianjin 300070, China; 2 300052 天津，天津市肺癌转移和肿瘤微环境重点实验室，天津市肺癌研究所，天津医科大学总医院 Tianjin Key Laboratory of Lung Cancer Metastasis and Tumor Microenviroment, Tianjin Lung Cancer Institute, Tianjin Medical University General Hospital, Tianjin 300052, China

**Keywords:** miRNA, 肿瘤, 耐药性, miRNA, Tumor, Chemoresistance

## Abstract

微小RNA（microRNA, miRNA）是一类长约22 nt的非编码小分子RNA，在转录后水平上负性调节靶基因的活性。微小RNA在肿瘤的发生、发展中发挥着广泛的作用，包括肿瘤的进展、分化、转移和耐药等。化疗药物的耐药是临床肿瘤治疗的难题，如何克服肿瘤细胞的顺铂耐药性引起人们的广泛兴趣。越来越多的研究证明微小RNA在影响肿瘤耐药中发挥着重要的作用。本文将对微小RNA与肿瘤治疗尤其是顺铂治疗的研究进展作一综述。

## Abstract (en)

MicroRNAs (miRNAs) are a class of small, about 22 nucleotides endogenous noncoding RNAs that negatively regulate their target genes' expression through post-transcriptional suppression. MicroRNA has a broad effect on tumorigenesis and tumor biological processes, including tumor development, differentiation, metastasis and chemoresistance. Chemoresistance represents a major obstacle in effective clinical treatment of tumors, how to conquer the chemoresistance in tumor arouses people's interst. A growing number of studies have proved that microRNAs play a crucial role in tumor chemoresistance. This view will discuss the the progress research between microRNA and tumor therapy, especially the cisplatin chemotherapy in tumor.

微小RNA（microRNA, miRNA）是一种内源性的非编码的RNA序列，一般由21-23 nt组成单链RNA分子，广泛存在于各种生物体中^[1]^，通过对靶miRNA的直接分解或抑制其翻译的方式在转录后水平调控基因的表达^[2]^，RNAase Ⅲ超家族成员Drosha剪切pri-miRNA形成pre-miRNA，后者进而被Dicer剪切形成成熟的miRNA^[3]^。

## 1 微小RNA在肿瘤中的生物学功能

1993年Lee等^[4]^在酵母中发现lin-4和let-7失活使上皮细胞倾向于分裂而不是正常分化，提示miRNA具有重要的生理功能。2002年Calin等^[5]^首次在慢性淋巴细胞白血病（chronic lymphocytic leukemia, CLL）中发现has-miR-15a和has-miR-16-1下降或缺失，确立了miRNA与肿瘤的相关性。miRNAs参与生理、病理过程，在肿瘤发生和发展中，不同的miRNAs分别发挥抑制肿瘤（tumor suppressor miRNA, TSmiRs）或促进肿瘤作用（oncogenic miRNAs, oncomiRs）^[6]^。过去的研究^[7]^表明miRNA基因表达的改变几乎与人类大多数恶性肿瘤病理形成有关，引起基因表达改变机制包括：缺失、扩增、miRNA位点突变、表观遗传基因沉默、以特定miRNAs为靶点的转录因子调节的紊乱等。Volinia等^[8]^通过进行大规模的肿瘤miRnome分析，确定了一些肿瘤相关的miRNA，例如miR-17-5p、miR-20a、miR-21、miR-92、miR-106a以及miR-155等，它们通过调节一些重要的癌基因或抑癌基因表达影响肿瘤发生。人们相继发现microRNA参与人体多种生命活动，可以促进细胞增殖^[9]^、对细胞的凋亡通路进行调节^[10]^、参与细胞周期的调控^[11]^、参与人体的代谢过程^[12, 13]^等。MiRNA不仅参与细胞增殖、分化、凋亡、周期调控等，对肿瘤的化疗耐药也具有重要作用。有研究表明miRNA与肿瘤转移有关。Nicoloso等^[14]^发现同种的miRNA不仅使特定肿瘤细胞具有侵袭能力，而且使肿瘤干细胞表现出特定表型，提示了在肿瘤起源和最后形成的机制之间的联系。

Calin等^[15]^研究证实了miRNA，特别是肿瘤相关miRNA在染色体上的特殊性，即肿瘤相关miRNA经常定位于一些染色体高变区域，可能导致LOH，缺失以及扩增等，从而引起miRNA表达变化或突变、缺失等，影响下游肿瘤相关基因表达，导致肿瘤发生。他还阐述了miRNA表达谱在肿瘤诊断、预后以及治疗效果监控方面的应用^[16]^。Yu等^[17]^筛选出胰腺癌细胞中高表达的miRNA，并研究发现miR17-5p与患者预后差有关，并能促进肿瘤细胞增殖和侵袭。Iorio等^[18]^通过基因芯片和Northern blot分析发现肿瘤组织中总miRNA表达与正常组织明显不同，差异表达的miRNA与某些乳腺癌生物病理特征有关，如雌激素和孕酮受体的表达、肿瘤分期、血管侵袭、增殖指数。

Mavrakis等^[19]^应用无偏倚的miRNA文库筛选（unbiased miRNA library screen）的方法，系统研究了急性T淋巴细胞白血病（T-ALL）相关的miRNAs，发现5个miRNAs（miR-19b、miR-20a、miR-26a、miR-92以及miR-223）在T-ALL发病中起到关键作用，并且这些miRNAs在T-ALL发生过程中抑制主要抑癌基因（例如*IKZF1*、*PTEN*、*BIM*、*PHF6*、*NF1*、*FBXW7*等）表达的作用相互重叠，相互协调。该研究揭示了T-ALL发生中microRNA-肿瘤抑制基因作用的新模式。

细胞耐药性是肿瘤化学治疗中的难题，关于miRNAs与肿瘤细胞耐药性的研究很多，也取得了一定的进展。Xia等^[20]^用miRNA芯片筛选出了人胃癌多药耐药细胞株中miRNAs的异常表达。miR-15b和miR-16在人胃癌多药耐药细胞株中表达下调，可以引起Bcl-2蛋白表达的增加，提示miR-15b和miR-16通过靶向作用于Bcl-2而导致胃癌细胞多药耐药性的形成，进而影响细胞凋亡。秦学博等^[21]^以吉非替尼敏感肺癌细胞PC9与吉非替尼耐药肺癌细胞PC9/AB11为细胞模型，初步筛选到了13个与肺腺癌吉非替尼耐药密切相关的miRNA。miRNA促进或逆转肿瘤细胞的化学药物耐药性。

## 2 顺铂耐药机制研究

顺铂临床应用于实体瘤中，有较广的抗癌谱，是已知的最有效的抗瘤药物之一。顺铂耐药限制了以顺铂为基础的化疗，顺铂耐药性的产生机制可能包括：药物吸收减少、药物灭活增加、DNA加合物修复增强。这些产生耐药的药理学机制起源于分子水平的改变，包括DNA损伤的识别、*HER*-*2*/*neu*基因的过表达、PI3-K/Akt通路的激活、p53功能的缺失、抗凋亡基因*bcl*-*2*的过表达、对细胞凋亡途径caspase途径的干扰等。通过研究，顺铂耐药的机制不断被人们发现。一系列的DNA修复序列表明铂类-DNA加合物的移除与人类A2780/C系列卵巢癌顺铂耐药有关。Ferry等^[22]^研究表明核酸切除修复的增强可导致细胞株的耐药，顺铂耐药被证实与对紫外线抵抗的增强有关，同时ERCC1的过表达也与顺铂耐药有关，进一步表明在顺铂耐药模型中，ERCC1-XPF核酸内切酶是核酸切除修复的决定性因素。内切修复交叉补体1（excision repair cross-complementing 1, *ERCC1*）基因表达水平与顺铂DNA加合物的修复有重要关联，有研究报道也与肿瘤细胞顺铂耐药水平有关。Chang等通过将ERCC1siRNA1和siRNA2转染细胞，发现ERCC1 mRNA和蛋白质表达均被明显抑制。在HeLa S3细胞中ERCC1表达的抑制导致顺铂诱导的DNA损伤修复活性的减低。从而为ERCC1siRNA应用于治疗肿瘤提供了新的发现^[23]^。MDR多药耐药性仍然是肿瘤化学治疗失败的主要原因之一，MDR1/P-gp过表达被认为是肿瘤细胞多药耐药的机制。Twist1与颈椎癌放疗耐受及化疗耐药有关，Zhu等^[24]^研究证实Twist1介导的MDR1/P-gp表达在颈椎癌细胞顺铂敏感性起重要作用，*Twist1*基因的沉默能下调MDR1/P-gp的表达。因此通过抑制Twist1表达来克服颈椎癌药物耐受可能成为颈椎癌治疗的新方法。卵巢癌患者用顺铂治疗一段时间后很容易产生对其的耐药性，Olivero等^[25]^研究表明肝细胞生长因子HGF可以增强顺铂治疗后卵巢癌细胞的死亡率。Holford等^[26]^通过卵巢癌细胞株体外实验发现具有空间位阻效应的铂类复合物AMD473可以使细胞逃避顺铂耐药。

## 3 MiRNA在肿瘤顺铂耐药的作用

肺癌是人类最常见肿瘤及死亡原因之一，目前公认的晚期非小细胞肺癌一线治疗标准是含铂的两药联合方案。以铂类为基础联合应用重组人血管内皮抑素或靶向化疗药物等方案被证实具有较好的疗效^[27]^。然而在使用顺铂化疗过程中，易产生顺铂耐药性，从而为肺癌患者化疗提出了难题。人们也在不断研究顺铂耐药机制并寻找克服或逆转顺铂耐药的方法。如Meng等^[28]^发现新城疫病毒NDV通过caspase途径可以诱导耐药肺腺癌细胞A549/DDP的凋亡，特别是caspase-9途径。同时MAPK通路及Akt途径也参与凋亡诱导过程。通过小鼠A549/DDP肺癌模型证实NDV有溶瘤作用。该研究从体外和体内试验研究表明NDV可以克服肺癌细胞的顺铂耐药。其中关于miRNA与肺癌顺铂耐药性的研究也很多。Galluzzi等^[29]^利用芯片技术来筛选人非小细胞肺癌顺铂耐药株A549/DDP中表达上调的miRNA。人工合成的靶向作用于miR-181a和miR-630的miRNA抑制剂作用于A549/DDP细胞后，pre-miR-181a和pre-miR-630表达增加，CDDP引发的细胞死亡减少。pre-miR-181a和pre-miR-630调节体内凋亡的线粒体/线粒体后途径，包括Bax寡聚核苷酸化、线粒体膜溶解、成熟的caspase-9和caspase-3的蛋白水解。另外，pre-miR-630阻断上游由于DNA损伤而引发继而引起p53激活的信号传导通路。该研究表明miR-181a和miR-630可以作为非小细胞肺癌对顺铂化疗的新型调节剂。

## 4 肺癌中miRNA的研究进展

MiRNA促进肺癌转移。O’Donnell等^[30]^发现，miR-17-92与小细胞肺癌中高表达的癌基因*c*-*Myc*相关，*c*-*Myc*基因过表达引起了miR-17-92的高表达。miR-17-92能加快癌细胞的生长，但其在肺癌发生发展中的作用机制尚不清楚，根据对其靶点预测其机制可能是通过抑制两个抑癌基因*PTEN*（phosphatase and tensin homolog）和*Rb2*而发挥其促进肿瘤作用。miR-21的靶点已被证明是多个肿瘤抑制基因的mRNA，包括*PTEN*、*PDCD*（programmed cell death）、*TPM1*（tropomyosin 1）和*SerpinB5*（serpin peptidase inhibitor, clade B member 5）基因，这些基因不仅对癌细胞恶性转化起负调控作用，而且能够抑制细胞运动和侵袭^[31-34]^。

MiRNA抑制肺癌的转移。let-7 miRNA家族是典型的具有抑癌作用的分子，其表达水平在多种肿瘤中明显下调，Ras和Myc的mRNA 3’UTR区有多个let-7结合位点，let-7靶向结合在其上导致翻译抑制，从而负向调控二者的表达；相反，let-7的减少必然导致相应蛋白的过表达，引起细胞转化，进而导致肿瘤的发生和发展^[35]^。miR-125b-1定位于11q23-24杂合型缺失（loss of heterozygosity, LOH）的微小区域，这一区域在肺部肿瘤、乳腺癌和卵巢癌中存在突变或缺失，提示miR-125b-1发挥了抑癌基因的作用。有研究表明，肺癌组织中miR-125b-1基因突变与肺癌患者的临床病理特征相关，且该基因突变与淋巴结转移之间存在正相关，表明miR-125b-1功能异常能增加患者发生淋巴转移的风险性，从而可能在肺癌细胞的转移过程中起重要作用^[18]^。

MiRNA调节肺癌细胞表型转化。Korpal等^[36]^报道，下调miR-200c表达可促进EMT发生，提高肿瘤细胞侵袭转移能力；而过表达miR-200c可抑制EMT发生，降低肿瘤细胞侵袭力。上皮细胞-间充质细胞转换（epithelial-mesenchymal transition, EMT）是指上皮细胞通过特定程序转化为具有间质表型细胞的生物学过程。通过EMT，上皮细胞失去细胞极性以及与基底膜连接等上皮表型，获得了具有较高迁移与侵袭、抗凋亡和降解细胞外基质等能力的间质表型。EMT是上皮细胞来源的恶性肿瘤细胞获得迁移和侵袭能力的重要生物学过程。

针对肿瘤患者循环系统中miRNA的研究已经开始，为研究肺癌转移过程中miRNA的作用开辟了一条新的道路。Rabinowits等^[37]^发现肺癌病人血液循环中的总肿瘤外切体和miRNAs水平与正常人有明显的不同，然而肺癌患者与健康人之间血液循环中的miRNA外切体及具有促肿瘤形成的miRNA种类相类似。提示可以将血液循环中的miRNA外切体作为肺腺癌的筛选试验。

## 5 MiRNA的前景展望

MiRNA可调控约三分之一的蛋白编码基因的表达，影响着多条信号传导通路。miRNA在不同肿瘤组织中表达水平的研究和分析，不仅有利于阐明肿瘤的发生发展机制，更为肿瘤的诊断和治疗提供了理论依据。随着对miRNA与肿瘤耐药性的研究进展及发现，以miRNA为靶点设计新的药物将为阻断肿瘤转移、改善患者预后提供有效的治疗途径。
